# How does leaders' information-sharing behavior affect subordinates' taking charge behavior in public sector? A moderated mediation effect

**DOI:** 10.3389/fpsyg.2022.938762

**Published:** 2022-12-07

**Authors:** Jun-Na Liu, Yun-Zhang Hou, Jun Wang, Ping Fu, Cong-Zhen Xia

**Affiliations:** ^1^School of Public Affairs and Administration, University of Electronic Science and Technology of China, Chengdu, China; ^2^School of Management, Fudan University, Shanghai, China; ^3^Business School, Huaqiao University, Quanzhou, China; ^4^Management School, Hainan University, Hainan, China; ^5^School of Management, University of Sanya, Hainan, China; ^6^Faculty of Engineering, University of Sydney, Sydney, NSW, Australia

**Keywords:** information sharing behavior of public sector leaders, taking charge behavior, public service motivation, emotional trust, moderated median model

## Abstract

**Introduction:**

Taking charge behavior (TCB) of civil servants is an important part of individual innovation performance, which is not only a key step for innovation in the public but also a real need for high-quality cadres construction in the public sector in the new era. Therefore, it is necessary to carry out an in-depth discussion on civil servants' taking charge behavior. Based on the theory of planned behavior, this paper constructs the framework of"cognition-motivation-behavior" to deeply explore the relationship between public sector leaders' information-sharing behavior and subordinates' taking charge behavior, as well as the mediating and moderating effects of subordinates' public service motivation and emotional trust.

**Method:**

This study collected 200 civil servants' questionnaires by online survey, and conducted regression analysis through SPSS/AMOS/PROCESS.

**Result and discussion:**

The empirical study finds that the information-sharing behavior of leaders in the public sector can significantly affect the TCB of subordinates; the public service motivation partially mediates the relationship between them; emotional trust positively moderates the mediation effect of public service motivation in the relationship between leaders' information-sharing behavior and subordinates' TCB in the public. This study not only enriches the research on civil servants' TCB theoretically but also provides meaningful enlightenment for promoting civil servants' taking charge behavior.

## Introduction

George Fredrickson highlighted that if one word can sum up the characteristics of public management in the late 20^th^ century and the early 21^st^ century, that is, “change” (Frederickson et al., 1999). People began to use words such as reengineering, reconstruction, innovation, and entrepreneurship to describe public management. Government innovation has been widely concerned and highly valued by governments around the world (Kamarck, 2003). To respond to the era of volatile, uncertain, complex, and ambiguous (VUCA), innovation in the public has always been an important strategy (Hansen and Pihl-Thingvad, 2019). In the past decades, changes and reforms in the public sector have taken place continuously (Fattore et al., 2018), and the public servants' taking charge is the first step of public sector innovation (Bysted and Hansen, 2015). Although reform projects are usually initiated by administrative officials or decision-makers in a top–down manner, or assigned by political orders, the actual reforms are all accepted by the middle-level and subordinates in the public (Ahmad et al., 2020; Hassan et al., 2021). Studies have found that civil servants are important initiators of innovation within government organizations and play an important role in government reform and innovation (Damanpour and Schneider, 2009). Middle-level and front-line managers in the public sector are the most common initiators of governance innovation in federal countries, such as the United States (Borins, 2000). Moreover, civil servants will innovate policy implementation, service supply, organizational processes, and affair concepts in the form of working groups (Torugsa and Arundel, 2015). Since the 18th National Congress of the Communist Party of China, Party, and government have put forward some requirements for civil servant groups, such as “to reform and innovate,” “to enhance the ability of reform and innovation,” “be full of pioneering and innovative spirit,” and “accurately recognize changes, scientifically adapt to changes, and actively seek changes” (Liu et al., 2015). Therefore, it is particularly important to conform to the actual needs of innovation in the government, stimulate the innovation motivation of the civil servants, and focus on the taking charge behavior (TCB) of individual civil servants.

Previous studies have verified that different types of leadership behaviors positively influence subordinates' TCB, such as empowered, participatory, transformative, shared, service-oriented, humorous, and parental leadership facilitating subordinates' TCB (Hackman and Oldham, 1976; Wu and Wu, 2007; Kim et al., 2015; Pundt, 2015; Ge, 2016; Tian and Sanchez, 2017; Hao and Long, 2020; Tang and Fang, 2020; Zhang et al., 2020). Among them, empowered leaders enhance subordinates' innovation competence and freedom by sharing performance goals, information, and other management practices (Fernandez and Moldogaziev, 2013; Demircioglu, 2018). Ethical leaders express ethical expectations to their subordinates and promote the subordinates to innovate for the benefit of the organization and the masses (Hassan, 2015). Transformational leaders enhance the sense of innovation and stimulate innovative behaviors of subordinates by maintaining high-quality communication and empowering them in participating in decision-making efficiently (Vigoda-Gadot and Beeri, 2012; Vandervoet, 2016). Servant leadership focuses on a strong bonding between the leaders and followers. It aims at providing valuable directions and support for followers to reduce the threats related to innovative work behavior and to promote the improvement of employees' innovative ability (Zada et al., 2022a). It can be found that, through information transmission, leaders have frequent interactions with subordinates, which promotes the subordinates' TCB. Although existing studies have examined the influence of leadership behavior on subordinates' TCB, the research on specific leadership information-sharing behaviors (ISB) on subordinates' TCB is very limited. Therefore, this study attempts to explore the influence of leadership behavior on subordinate behavior in the public sector and tries to answer the question of whether the relevant research confirmed in the field of enterprise management is also applicable in the public sector. Moreover, most of the abovementioned studies have been explored in the private sector and are rarely seen in the public sector. Nowadays, affected by the “New Public Management Movement,” new public administration theories and management models reflect the application of advanced management theories and practices of the private sector to the public sector, which aims at improving the efficiency of the government sector (Li, 2004). Then, taking ISB into the public sector, exploring whether the public sector leaders' ISB can inspire their subordinates' TCB is also worthy to be researched.

When we are studying the influence of public sector leaders' behaviors on subordinates' behaviors, public service motivation (PSM) is a mediated variable that cannot be ignored. PSM refers to the psychological tendency of individuals in responding to the goals of the public sector (Perry and Wise, 1990) and is a special inner motivation that transcends individual interests and zealously serves the public, nation, country, and mankind (Liu et al., 2015). People with high PSM tend to work in the public sector and are more willing to give full play to their discretion for public service, and then make changes in public service. Therefore, this research tries to identify the mediating variable of PSM between the government leaders' ISB and the subordinates' TCB. In addition, leaders in the public sector not only share work information but also share non-work information in open communication and interaction with their subordinates, which is conducive to the establishment of emotional trust (ET) between leaders and subordinates and forming high-quality leader–follower relationships (Zada et al., 2022b). ET is based on mutual interaction and attraction, deepened through long-term and frequent exchanges and communication between individuals and is manifested as a concern for the welfare of the trusted person (Zhang et al., 2015). In the public sector, when subordinates have strong ET, their TCB can be affected differently by their leaders' behavior. Therefore, this study attempts to test how ET moderates the relationship between the ISB of government leaders and the TCB of subordinates.

To sum up, this study intends to introduce the relationship between leaders' ISB and subordinates' TCB in the private sector into the public sector from the perspective of new public management. Based on the theory of planned behavior, this study explores the relationship between the ISB of government leaders and the TCB of subordinates and further discusses the mediated effect of PSM and the moderated effect of ET in the relationship between them. Theoretically, this study introduces leaders' ISB into public sector management, attempting to test the positive effect of public sector leaders' ISB on subordinates' TCB, which enriches the research on the influencing factors of civil servants' TCB. Second, this study attempts to examine the mediating effect of PSM in the relationship between leaders' ISB and subordinates' TCB, which explains the key links of public sector leaders' ISB influencing subordinates' TBC and the influence mechanism of civil servants' TCB more clearly. Third, this study explores the possible moderating effect of subordinates' ET on the relationship between the ISB of government leaders and the PSM of subordinates, which also expands boundary conditions between the above factors. Practically, this study discusses and verifies the internal mechanism and boundary conditions of how government leaders' ISB affects their subordinates' TCB, which provides important evidence for strengthening leaders' ISB, cultivating civil servants' PSM, and promoting civil servants' TCB. It also supplies some practical suggestions for public sector innovation and civil servants' training.

## Theory and hypotheses

### Theory of planned behavior

The theory of planned behavior is a well-known attitude–behavior relationship theory (Duan and Jiang, 2008), which is used to explain human behavior in specific situations (Ajzen, 1991). This theory believes that behavioral attitudes, subjective norms, and perceived behavioral control determine behavioral intentions, and behavioral intentions directly determine actual behaviors. Behavioral attitude is an individual's like or dislike, positive or negative evaluation of engaging in a specific behavior, and the core of which is a behavioral belief. Subjective norms refer to the social pressure individual experiences when deciding whether to engage in a specific behavior, reflecting the influence of important others or groups on the individual's behavioral decision-making. Perceptive behavioral control refers to the degree of difficulty an individual perceived in controlling and performing a certain behavior (Ajzen, 2011). The theory of planned behavior has been widely used to predict the rational and challenging behaviors of individuals in organizations, which also provides a theoretical perspective for understanding the pre-factors of the civil servants' TCB. In this study, we assume that civil servants are surrounded by uncertainty. If they want to change the status quo of work, there will be a denial of existing rules and procedures of the organization. Therefore, before implementing TCB, civil servants would be more cautious. It is a dynamic process from the ISB of the public sector leaders to subordinates' TCB. They have completed the transformation from being capable of change, willing to change, to making change. First, when leaders in the public sector share information, their subordinates not only receive ample information resources but also feel the goodwill of leaders. Second, when subordinates realize that the leader's ISB is a kind of trust and recognition for them, they will be encouraged to change the status quo of work, which nurtures the motivation of TCB. Finally, subordinates with a high willingness of TCB will implement change behaviors, externalizing the motivation of change into behaviors of change. Therefore, this research constructs a theoretical framework of “cognition-motivation-behavior” based on planned behavior theory, which is used to analyze how the public sector leaders' ISB affects their subordinates' TCB.

### Leaders' ISB and subordinates' TCB in the public sector

Leader's ISB refers to leaders sharing views and opinions on various topics with their subordinates proactively (Nifadkar et al., 2019). Previous studies have more defaulted it as the leader's work ISB (Hatfield and Huseman, 1982; Snyder and Morris, 1984). Nifadkar et al. (2019) divided leaders' ISB into work ISB and non-work ISB. The former refers to leaders' proactive actions aiming at sharing information with subordinates that may help them complete official tasks, clarify official policies, explain performance expectations, and inform them of the procedures to fulfill their official responsibilities. The latter refers to leaders' communication with subordinates about their concerns, interests, and activities outside of the organization. The more frequently information sharing takes place, the more tasks, goals, and policies leaders communicate with their subordinates, and the more interests, hobbies, and activities outside the organization leaders convey (Chen et al., 2018). As a positive leadership behavior, ISB has strengthened the connection with subordinates and further enhanced the organizational LMX. In this context, subordinates will easily believe that they are valuable and trustworthy (He et al., 2020), which encourages them to participate in the development and progress of the organization with a master mentality (Gao et al., 2011). In this study, we defined the public sector leaders' ISB as leaders communicating the goals and policies of the government, explaining relevant decisions, and sharing personal interests, as well as family conditions to subordinates through open communication in the process of public affairs governance.

Taking charge behavior is defined as a behavior that voluntarily makes constructive efforts to optimize work processes and improve work methods, aiming at organizational functional changes. It can also be called transformational organizational citizenship behavior (Homberg et al., 2019; Lin and Zhang, 2019). This study defines civil servants' TCB as the behavior of civil servants who actively propose and share new ideas and use or promote new methods around working methods, working procedures, and policy requirements. That is, to improve the effectiveness of policy implementation and enhance the performance of public services, civil servants take proactive and constructive changes in working methods, policies, and procedures (Vigoda-Gadot and Beeri, 2012; Tan, 2019). TCB is regarded as a risky behavior that challenges the status quo. Except for individual's characteristics, TCB has also been affected by organizational contexts such as leadership behavior (Bettencourt, 2004).

Measures taken by different types of leadership styles in creating an organizational atmosphere or role demonstrating would encourage civil servants' TCB (Meijer, 2014). This study believes that leaders' ISB will encourage subordinates' TCB in the public sector or civil servants' TCB. Specifically, in the public sector, leaders with high ISB will explain organizational tasks to subordinates to help them understand how their work integrated with sectors' goals, clarify their own roles and responsibilities (Guo and Liao, 2014), enhance autonomy and responsibility, and improve awareness of the importance of their work, which stimulates subordinates' TCB. Moreover, in the process of information sharing, the trust between leaders and subordinates is bound to increase. When subordinates obtain more trust and support from the leader, they would be more confident in trying new things (Chiaburu and Baker, 2006). Because the leader in the public sector would tolerate and understand some wrong experiences of subordinates, which is of great intensive for subordinates and then inspires their TCB. In addition, when leaders share information with subordinates in the public sector, it will also stimulate the subordinates' reciprocal exchange psychology and show the same information exchange behavior (Chan, 2014; He et al., 2021). The accumulation of information provides sufficient resources for the subordinates to take charge. Different types of leaders provide subordinates with material or non-material work resources, and subordinates repay the leader with behavioral innovation (Wynen et al., 2020). Leaders' ISB in the public sector could be regarded as providing information resources for subordinates, which would promote subordinates' TCB.

Based on the above statements, we propose the following hypothesis:

H1: Leaders' ISB in the public sector positively affects the subordinates' TCB.

### Mediation effect of PSM on the relationship between leaders' ISB and subordinates' TCB in the public sector

#### Leaders' ISB and subordinates' PSM in the public sector

In Perry and Wise (1990), first proposed the concept of PSM and developed the theory of PSM. PSM includes four dimensions: attraction to policy-making, commitment to the public interest, compassion, and self-sacrifice (Perry, 1996). In the context of Chinese culture, some scholars have summarized it into three dimensions: attraction to participate in public decision-making, identification with public interests, and dedication and sacrifice (Liu et al., 2018). In this study, PSM means the internal motivation of civil servants to provide services for the public in engaging in public affairs.

Public service motivation is not invariable, but variable with the change in the external environment and internal cognition (Wang and Shu, 2018). Existing studies have verified that social and historical background, organizational environment, individual characteristics, and behavior as independent variables would affect the formation of PSM (Perry, 2000). Leadership factors, as an important variable of organizational environment, undoubtedly play an irreplaceable role in the formation of subordinates' PSM (Bass, 1985; Liu, 2015), especially transformational leadership, service-oriented leadership, ethical leadership, and relational behavior of leaders will positively affect subordinates' PSM (Chen and Lin, 2016; Ge, 2016; Tang and Fang, 2020; Chen and Liu, 2021). However, the relationship between leaders' ISB, specific leadership behavior, and subordinates' PSM still needs to be further explored.

Based on the influence of “guanxi” culture with a “diversity-orderly structure,” leaders' ISB to subordinates will shorten the distance between leaders and subordinates, create a trustworthy working environment, and form a high-quality relationship between leaders and subordinates through open exchanges and communication. Under these conditions, the basic psychological needs of subordinates can be fully satisfied, thus motivating them to pursue higher-level values, such as the mission of serving others and society (Perry et al., 2010; He et al., 2019). Li and Wang (2016) believes that a good relationship with leaders could enhance subordinates' PSM. Leaders' ISB in the public sector is also conducive to establishing a good relationship between leaders and subordinates, which would promote the formation of subordinates' PSM. In addition, an individual sense of security and belonging is an effective environmental factor of individual internal motivation (Zhang et al., 2010). Leaders' ISB in the public sector also includes sharing interests with subordinates and helping clarify policy objectives and tasks, which creates a relaxed working atmosphere and relieves the spiritual height of tension and stress. In this way, subordinates' sense of security and belonging would be strengthened, then, promoting the public service motivation of subordinates.

Based on the above statements, we propose the following hypothesis:

H2: Leaders' ISB in the public sector positively affects the subordinates' PSM.

#### Subordinates' PSM and TCB in the public sector

Public service motivation has a significant influence on subordinates' attitudes and behaviors in the public sector. Some studies have shown that civil servants' PSM positively influences the change in organizational citizenship behavior (Chen and Lin, 2016), TCB (Homberg et al., 2019; Chen and Liu, 2021), innovation behavior (Tan, 2019), etc. Moreover, PSM is an important motive force for the innovation of civil servants (Miao et al., 2018). This study also believes that subordinates' PSM would promote their TCB in the public sector. Specifically, the stronger the subordinates' PSM in the public sector is, the more they will show the value tendency and altruistic behavior tendency of serving society (Chen et al., 2019). They would insist on the faith of serving people and pursuing the tenet of serving the people. Thus, subordinates in the public sector are required not only to meet basic job requirements but also to optimize work processes and methods, which are used to change the status quo and bring functional changes to the organization (Li et al., 2019).

Subordinates in the public sector with high-level PSM will identify their selfless service role more clearly, integrating the sense of responsibility and mission of serving the people into their work, challenging the status quo of work, making changes, and practicing more proactive reform behaviors (Chen and Lin, 2016). At the same time, they will explore how to respond to public demand and serve public interests better by using the discretionary space, thus presenting more TCB. In addition, subordinates in the public sector with strong PSM are full of the spirit of sacrifice for the public interest, and they are willing to bear the risks and other negative effects brought by innovation. As a result, they dare to innovate and reform (Tan, 2018). Relevant literature also proved that the stronger PSM of subordinates is, the less resistant they are to taking change (Homberg et al., 2019).

Based on the above statements, we propose the following hypothesis:

H3: Subordinates' PSM positively affect their TCB in the public sector.

#### Mediated effect of PSM in the relationship between leaders' ISB and subordinates' TCB in the public sector

From the above argument, we believe that leaders' ISB in the public sector positively influences the subordinates' TCB. Furthermore, many literature studies take PSM into account in studying the relationship between leaders' ISB and subordinates' TCB. Considering the public nature of government departments, leaders usually convey the importance of public service to their subordinates to cultivate their motivation to actively provide public service to citizens, which also accords with the role positioning of people's public servants. According to the theory of planned behavior, when leaders share information with subordinates, subordinates will recognize leaders' trust, at the same time, receive the value concept of public service conveyed by leaders, and then strengthen subordinate's PSM of serving citizens and society, thus promoting subordinates' TCB, which reproduces the progress cognition—motivation—behavior.

Moreover, public service motivation is a behavioral tendency accompanied by positive emotional experience, which is an important link between the external environment and employee behavior (Chen and Wu, 2008; Guo et al., 2014). This study believes that in the public sector, civil servants' PSM is an important motivation that can effectively receive the information shared by leaders and make positive responses. High PSM can make up for the lack of external motivation (Miao et al., 2018) and form a strong sense of responsibility, mission, self-sacrifice spirit, etc. These elements can inspire civil servants to actively participate in the decision-making process of government departments and dare to put forward new ideas, which promotes the generation of TCB. In short, leaders' ISB in the public sector would influence subordinates' TCB through the intermediary variable of PSM.

Based on the above statements, we propose the following hypothesis:

H4: PSM mediates the relationship between leaders' ISB and subordinates' TCB in the public sector.

### Moderated effect of emotional trust

Subordinates' trust in the leaders can be described as a psychological state in which they are willing to expose weaknesses to the leader based on positive expectations of the leader's intentions and behaviors without fear of being taken advantage of by the leader (Wei and Long, 2011). ET is built on the emotional interaction between subordinates and leaders, and the positive reciprocal experience and frequency of interaction will affect its formation (Mcallister, 1995). Existing studies have verified that ET is a special and deep psychological state, and once formed, it would have a lasting and stable influence on subordinates (Miao et al., 2014). Leaders' behavior also has a great influence on the formation of subordinates' trust (Chen et al., 2014), and leaders' ISB shows their trust to subordinates to great extent (Shi et al., 2012). We believe that leaders' ISB in the public sector will also produce subordinates' trust to varying degrees. As time goes by, this psychological state will affect subordinates' motivation to serve the public. Therefore, this study focuses on the moderated effect of subordinates' ET.

Subordinates with high ET have a positive attitude toward the leaders' ISB, and they regard leaders' ISB as leaders' expressions of trust and affirmation. In this situation, leaders' ISB makes subordinates strongly feel cared for and concerned by their leaders, which stimulates subordinates' sense of responsibility and mission, improves their enthusiasm, and promotes the formation of PSM. However, subordinates with low ET will hold a negative attitude toward the leader's ISB, regarding it as pressure from leaders. In this situation, leaders' ISB would cause subordinates' fear and exacerbate the sense of distance between leaders and subordinates, which reduces subordinates' sense of identity and responsibility to the organization and restrains the formation of PSM. Therefore, we assume that the level of ET could affect the mechanism of leaders' ISB and subordinates' PSM. That means in the high-level ET, leaders' ISB promotes the formation of subordinates' PSM; in the low-level ET, leaders' ISB restrains the formation of subordinates' PSM.

Based on the above statements, we propose the following hypothesis

H5: ET moderates the relationship between leaders' ISB and subordinates' PSM in the public sector.

Based on the above hypotheses, subordinates' PSM mediates the relationship between leaders' ISB and subordinates' TCB, while subordinates' ET moderates the relationship between leaders' ISB and subordinates' PSM. Therefore, we propose that ET moderates the mediation effect of PSM on leaders' ISB and subordinates' TCB in the public sector, namely, a moderated mediation effect. Specifically, in the condition of high ET, leaders' ISB in the public sector would significantly promote subordinates' PSM and then lead them to take charge. On the contrary, in the low-level ET, leaders' ISB affects subordinates' PSM less and then weakens the mediation effect of PSM on the relationship between leaders' ISB and subordinates' TCB.

Based on the above statements, we propose the following hypothesis:

H6: ET moderates the mediation effect of PSM on the relationship between leaders' ISB and subordinates' TCB.

**Figure 1:**
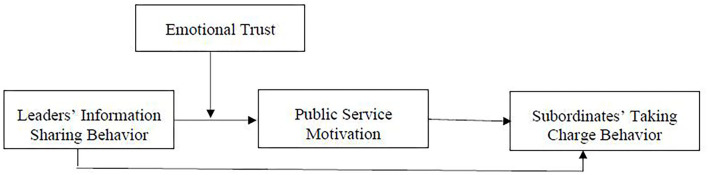
Conceptual model.

Thus, we construct the conceptual model as shown in Figure 1.

## Research design

### Research samples

The data for this study came from a questionnaire survey of civil servants in Shanghai, Shandong, Jiangxi, Guangdong, Guangxi, Xizang, and other provinces and cities in China. The respondents of the questionnaire come from administrative departments, judicial departments, party and mass organs, the National People's Congress, and other departments of China, which have good representativeness. In this survey, a total of 300 questionnaires were sent out and 203 were recovered, with a recovery rate of 67.67%. After eliminating 3 invalid questionnaires, 200 valid questionnaires were obtained, with an effective rate of 98.52%.

According to the descriptive statistics of samples, 52% are male participants and 48% are female participants; 65.69% are 25–35 years old, 19.12% are 25 years old or younger, 12.25% are 35–45 years old, 1.96% are 45–55 years old, and 0.98% are 55 years old or older; 69.12% of respondents are undergraduates, 23.04% are postgraduate, 6.86% are junior college students, and 0.98% are with doctoral degrees; 63.24% of respondents are staff members, 19.12% are section chiefs, 13.73% are clerks, 2.94% are division chiefs, and 0.98% are director general or above. The participants who are working 3 years and below account for 41.18%, 3–5 years account for 23.53%, 5–10 years account for 17.65%, 10–15 years account for 8.82%, and above 15 years account for 8.82%; 64.22% of respondents are in the eastern region, 26.96% of respondents are in the middle region, and 8.82% of respondents are in the western region. Respondents in government agency account for 59.8%, the party and the masses agency account for 23.04%, judiciary agency accounts for 16.67%, and 0.49% is in national peoples' congress or people's political consultative. According to the above data, most of the research respondents in this paper are young and highly educated civil servants, who are fresh out of school and are more agile in thinking. They are more likely to inspire PSM under the information sharing of leaders, thus leading to active reform, which is in line with the purpose of this study.

### Measure instruments

The main variables measured in this study are leaders' ISB in the public sector, subordinates' TCB, PSM, and ET. All measuring scales are derived from mature scales used in the relevant literature. Through the translation of multiple people, some items are revised to facilitate understanding and answers. Finally, a questionnaire is formed to investigate the influence of leaders' ISB in the public sector on subordinates' TCB.

We adopted the 12-item scale suggested by Nifadkar et al. (2019) to measure leaders' ISB in the public sector. In combination with the actual situation of the public sector, Nifadkar's scale (2019) was translated and modified to better measure leaders' ISB, items including “My leader will inform me of official regulations and policies” and “My leader will share his/her family plan with me.” The Cronbach's α value is 0. 935. We adopted the 9-item scale suggested by Vigoda-Gadot and Beeri (2012) and Homberg et al. (2019) to measure subordinates' TCB, items including “I will try to change the way of work to improve efficiency” and “I will try to correct imperfect/wrong work procedures or measures.” The Cronbach's α value is 0.963. We adopted the 8-item scale suggested by Bao and Li (2016) to measure subordinates' PSM, items including “It is important for me to make contributions to social welfare” and “I am willing to sacrifice my interests for social welfare.” The Cronbach's α value is 0.925. We adopted the 5-item scale suggested by Mcallister (1995) and Zhang et al. (2015) to measure subordinates' ET, items including “I and my leader can freely share thoughts/feelings and hopes.” Cronbach's α value is 0.936.

All items are measured on a 5-point Likert scale (1 = strongly inconsistent and 5 = strongly consistent). In addition, previous studies have shown that gender, age, educational level, working duration, and other factors would affect civil servants' TCB (Demircioglu, 2020). Therefore, the above variables are taken as control variables in this study.

## Data analysis

### Validity testing

First, the recovered data were used to take exploratory factor analysis by SPSS 22.0, the KMO value was 0.940, >0.7. Then, we took confirmatory factor analysis by AMOS 24.0 to verify the discriminant validity among the four variables, which are leaders' ISB in the public sector, subordinates' TCB, PSM, and ET. These variables were established factor models to compare their fittingness, the results are shown in Table 1. From Table 1, we can see that the four-factor model fits well, CFI and IFI values were 0.901, RMSEA was 0.085; *X*^2^/*df* was 2.440. According to these fitting indexes, we think the four-factor model is much better than those of other factor models and achieves a high standard.

**Table 1:** Result of confirmatory factor analysis.

**Model**	***X* ^2^**	***df***	***X*^2^/*df***	**RMSEA**	**CFI**	**IFI**
Four-factor model	1,239.714	508	2.440	0.085	0.901	0.901
Three-factor model	2,583.157	524	4.930	0.141	0.720	0.722
Two-factor model	3,426.474	526	6.514	0.166	0.606	0.608
Single factor model	4,457.643	527	8.459	0.194	0.466	0.469

Four-factor model: leaders' ISB, ET, PSM, and subordinates' TCB.

Three-factor model: leaders' ISB + ET, PSM, and subordinates' TCB.

Two-factor model: leaders' ISB + ET + PSM and subordinates' TCB.

Single factor model: leaders' ISB + ET + PSM+ subordinates' TCB.

### Common method bias analysis

In this study, Harman's single-factor test was used to test the common method bias between variables. The test results showed that there were 5 factors with an eigenvalue >1, and the total variance interpretation was 77.2%. The variance interpretation of the first principal component was 18.784%, less than half of the total variance interpretation. Thus, common method bias was not so serious in this study.

### Descriptive statistics analysis

We carried out the descriptive statistics and correlation statistics of each variable through SPSS, and the results are shown in Table 2. In Table 2, we can see that leaders' ISB in the public sector is positively correlated with subordinates' TCB (*r* = 0.778, *p* < 0.01), ET (*r* = 0.429, *p* < 0.01), and PSM (*r* = 0.450, *p* < 0.01). ET is positively correlated with subordinates' TCB (*r* = 0.509, *p* < 0.01) and PSM (*r* = 0.476, *p* < 0.01). PSM is positively correlated with subordinates' TCB (*r* = 0.725, *p* < 0.01). Based on these results, we could verify the hypotheses proposed further.

**Table 2:** Mean, standard deviations, and correlations of variables.

**Variables**	**M**	**SD**	**1**	**2**	**3**	**4**	**5**	**6**	**7**	**8**	**9**	**10**	**11**
1. ISB	2.908	1.838	1.000										
2. ET	3.270	2.208	0.778**	1.000									
3. PSM	4.113	1.502	0.429**	0.476**	1.000								
4. TCB	4.058	1.580	0.450**	0.509**	0.725**	1.000							
5. Gender			−0.027	−0.024	0.025	0.030	1.000						
6. Age			−0.089	−0.112	−0.072	−0.068	−0.166	1.000					
7. Educational level			−0.004	−0.016	−0.127	−0.108	0.122	0.041	1.000				
8. Position level			−0.124	−0.120	−0.124	−0.089	0.002	0.454**	0.252**	1.000			
9. Working duration			−0.071	−0.103	0.042	−0.020	−0.181	0.744**	−0.095	0.509**	1.000		
10. Region			−0.130	−0.121	−0.085	−0.001	0.012	0.137	−0.074	0.129	0.168*	1.000	
11. Nature of position			−0.123	−0.119	−0.021	−0.057	0.047	0.001	−0.017	−0.087	−0.056	0.056	1

** means p < 0.01; * means p < 0.05; two-tail testing is conducted; ISB = leasers' information-sharing behavior in the public sector; ET = emotional trust; PSM = public service motivation; TCB = subordinates' taking charge behavior.

### Mediation effect test

We test the mediation effect of PSM on leaders' ISB in the public sector and subordinates' TCB through PROCESS suggested by Hayes (2013), and the results are shown in Table 3. In model 3, having controlled gender, age, education background, working years, region, and post nature, we found that leaders' ISB in the public sector positively affects subordinates' TCB (β = 0.389, *p* < 0.001), thus, H1 is verified. In model 1, leaders' ISB in the public sector positively affects subordinates' PSM (β = 0.343, *p* < 0.001), thus, H2 is verified. In model 4, we take leaders' ISB and PSM as independent variables and compared with model 2, the results show that the regression coefficient of subordinates' TCB decreased from 0.389 to 0.153, *p* < 0.001, and PSM positively affects subordinates' TCB (β = 0.687, *p* < 0.001). Therefore, H3 and H4 are verified. At the same time, we use the Bootstrap method to test the mediation effect of PSM. In Table 4, the results show that the indirect effect value is 0.236, accounting for 60.64% of the total effect, and the 95% confidence interval is [0.142, 0.338], excluding 0. It further verifies the partial mediation effect of PSM between leaders' ISB in the public sector and subordinates' TCB.

**Table 3:** Testing of mediation effect and moderation effect.

**Constant**		**PSM**		**TCB**	
		**Model 1**	**Model 2**	**Model 3**	**Model 4**
	Gender	6.281	7.760	5.620	1.306
Control variable	Age	−0.337	−0.326	−0.268	−0.037
	Educational level	−0.220	−0.196	−0.225	−0.074
	Position level	−0.210	−0.251	−0.103	0.041
	Working duration	0.291*	0.278*	0.192	−0.008
	Region	0.108	−0.076	0.118	0.193
	Nature of position	0.055	0.056	−0.007	−0.045
Independent variable	ISB	0.343***	0.070	0.389***	0.153**
Mediated variable	PSM				0.687***
Moderated variable	ET		0.290***		
Interactive item	ISB*ET		0.057**		
*R* ^2^		0.230	0.313	0.230	0.558
*F*		7.112***	8.618**	7.111***	26.6489***

(1) ^***^Means p < 0.001; ^**^means p < 0.01; ^*^means p < 0.05; (2) regression coefficients in the table are non-standard coefficients.

**Table 4:** Mediation effect of PSM based on bootstrap.

	**Effect**	**SE**	**95% confidence interval**	
			**Lower limit**	**Upper limit**
Total effect	0.389	0.056	0.278	0.499
Direct effect	0.153	0.047	0.061	0.246
	**Effect**	**Boot SE**	**95% confidence interval**	
			**Boot** **Lower limit**	**Boot** **Upper limit**
Indirect effect	0.236	0.503	0.142	0.338

### Moderation effect test

Based on the results of model 1 in Table 3, leaders' ISB in the public sector positively affects subordinates' PSM. To test the moderation effect of ET on leaders' ISB in the public sector and subordinates' PSM, we added the interaction item of ET into model 2 and conducted a regression analysis. The results show that the regression coefficient of the interaction term is significant (β = 0.057; *p* < 0.05). Therefore, ET moderated the relationship between leaders' ISB in the public sector and PSM. Thus, H5 is verified. That is, when subordinates' ET is strong, the influence of leaders' ISB on subordinates' PSM would be significantly enhanced, whereas, when subordinates' ET is weak, the influence of leaders' ISB on subordinates' PSM would be significantly weakened.

**Figure 2:**
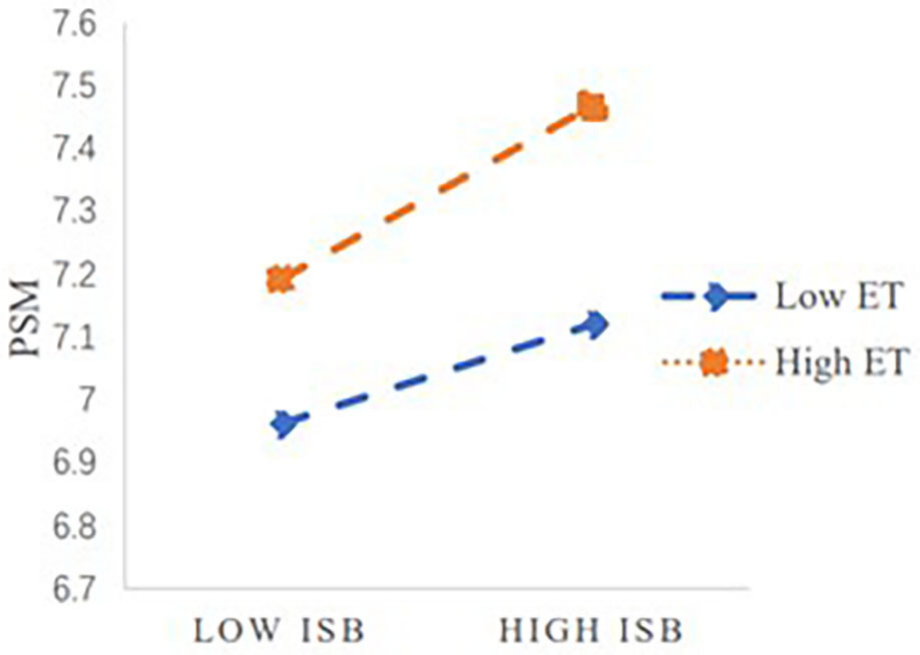
Moderation effect of emotional trust on the relationship between leaders' information sharing behavior in the public sector and subordinates' taking charge behavior.

To observe the moderation effect of ET more intuitively, this study drew the decomposition graph of the moderating effect according to the method recommended by Aiken and West (1991), as shown in Figure 2. Leaders' ISB in the public sector has different effects on subordinates' PSM in different levels of ET. When subordinates' ET is high, the slope of the line is steeper, and leaders' ISB in the public sector has a strong impact on subordinates' PSM. When subordinates' ET is low, the slope of the line is relatively gentle, and leaders' ISB in the public sector has a weak impact on subordinates' PSM.

### Moderated mediation effect test

When testing whether ET moderates the mediation effect of PSM on leaders' ISB and subordinates' TCB, we integrated relevant variables into a model for verification and analysis through PROCESS. Model 7 is selected, and the sample size of Bootstrap is set to 5,000. The confidence interval is set as 95%, and the running results are shown in Table 5. In Table 5, we can observe that ET significantly moderated the mediation effect of PSM on leaders' ISB and subordinates' TCB (the moderating index is 0.039, 95% confidence interval [0.005, 0.074], exclude 0), and there is a moderated mediation effect. When the ET level is low, the 95% confidence interval [−0.206, 0.121] includes 0, indicating that the mediation effect of PSM is not significant under the condition of low ET. When the ET level is high, the 95% confidence interval [0.034, 0.234] excludes 0, indicating that the mediation effect of PSM is significant under the condition of high ET. Therefore, H6 is supported.

**Table 5:** Mediation effect of PSM under different levels of ET.

**Moderated variable**	**Indirect effect**	**Boot SE**	**95% confidence interval**	
			**Lower limit**	**Upper limit**
High ET	0.134	0.051	0.034	0.234
Low ET	−0.038	0.083	−0.206	0.121
Moderated mediation index	0.039	0.018	0.005	0.074

## Research conclusion and discussion

### Research conclusion

Based on the theory of planned behavior, this study constructs a moderated mediation model of leaders' ISB in the public sector affecting subordinates' TCB under the framework of “cognition-motivation-behavior.” We introduce the research on the relationship between leaders' ISB and subordinates' TCB in Chinese government departments. Taking 200 Chinese civil servants as samples, the empirical research found that the leaders' ISB in the public sector positively affects subordinates' TCB. PSM mediated the relationship between them. ET moderated the relationship between leaders' ISB in the public sector and PSM. Moreover, ET moderated the mediation effect of PSM on the leaders' ISB in the public sector and subordinates' TCB.

With the development of the economy and society, government departments in the new era have higher innovation requirements for civil servants and leaders, calling for civil servants to take charge and promoting the construction of higher-quality government departments. TCB is reflected in the individual voluntary efforts to improve and update the existing workflow to realize the functional change of the organization. Leaders' ISB is stimulating subordinates' trust in leaders and recognition of the organization through the transmission of work information and non-work information to subordinates to spontaneously produce TCB. This study suggests that the leaders' ISB is naturally compatible with the subordinates' TCB, and it can significantly improve the subordinates' TCB. This process is also realized through subordinates' PSM. Moreover, when subordinates have a high degree of ET in leaders, and the organization, leaders' ISB is more likely to lead to TCB based on the PSM of subordinates.

### Theoretical significance

The theoretical contributions of this study include the following three aspects:

First, we enrich the research results of ISB and TCB in the field of public administration by introducing the relationship between them into the Chinese public sector. Previous studies on leaders' ISB are more reflected in the field of business management and verified its positive impact on employees' TCB (Zhu et al., 2021). This study took civil servants as research objects, detailing the factors of leaders' behavior that affects civil servants' TCB, empirically testing the influence of leaders' ISB in the public sector on subordinates' TCB, which enriches the study on antecedent variables of civil servants' TCB.

Second, we explore the influence mechanism of leaders' ISB in the public sector on subordinates' TCB, opening the “black box” from leaders' ISB to subordinates' TCB. From the perspective of the theory of planned behavior, this study constructed the theory model of “cognition-motivation-behavior,” more clearly showing the process of leaders' ISB in the public sector affecting subordinates' TCB through PSM, which enriches the application of the theory of planned behavior and broadens the research perspective of PSM.

Third, based on the Chinese culture, we examine the boundary effects of leaders' ISB and PSM on subordinates' TCB by introducing ET as a moderated variable. It also echoes Zhang and Zhou's (2014) suggestion that leadership behavior significantly impacts employee innovation in the condition of high trust. Therefore, we delineated a clear condition that leaders' ISB in the public sector affects subordinates' TCB by identifying the boundary condition of ET.

### Practical implication

Civil servants' TCB in their work is of primary importance in the public sector. How leaders in the public sector motivate subordinates' TCB is a major challenge in the field of public administration. The practical implication for civil servants' TCB provided by the conclusion of this study includes the following.

First, leaders in the public sector should enhance information sharing with their subordinates to stimulate subordinates' TCB. Civil servants' TCB is the spontaneous innovation of daily work processes and methods, which is the backbone of the reform and innovation in the public sector. The research of this study found that leaders' ISB in the public sector significantly promotes subordinates' TCB. As one of the important contents of leadership role behavior, information conveying (Xu and Ou, 2012) positively drives subordinates' TCB. With the development of information technology and the increasing knowledge mastered by subordinates, it is no longer appropriate for leaders to obtain power through blockading and monopolizing information and restricting subordinates' Nanjing University of Administration Research Group (2004). Subordinates increasingly need to get sufficient information to cope with the complex and changeable organizational environment (Chen et al., 2018). Therefore, leaders in the public sector should strengthen information sharing with subordinates, communicating with them as much as possible, and form a benign interactive relationship, so that subordinates can not only obtain more information resources but also establish trust and ownership with leaders, which enable subordinates to change and dare to change. The communication between leaders and subordinates can be conducted through regular symposiums, regular outdoor activities, dinner parties, and other leisure and entertainment, so that leaders and subordinates can not only exchange more information on work but also share information on life. Some leaders may selectively share information with subordinates based on their preference, which is not conducive to subordinates' TCB. Therefore, the leaders in the public sector need to share information with subordinates more actively, to provide resource support and emotional encouragement for subordinates' TCB.

Second, the public sector should pay attention to cultivating civil servants' PSM and do a good job in the psychological construction of seeking happiness for the people and development for society. This study shows that civil servants' PSM not only affects their TCB, but mediates the relationship between leaders' ISB and civil servants' TCB partially, which means civil servants' PSM is a key variable deserving more attention. It requires the public sector to pay attention to those of high PSM in the daily selection of civil servants, and at the same time, strengthen the cultivation of PSM for the current civil servants. Through more theoretical learning and practice, civil servants could deepen their perception of PSM, strengthen their attention to PSM, stimulate their TCB, and at the same time, effectively convey the influence of leaders' ISB in the public sector on subordinates' TCB.

Third, leaders in the public sector should attach importance to the establishment of subordinates' ET, forming a good relationship between leaders and subordinates, and constructing a harmonious organizational atmosphere, which creates conditions for subordinates to take charge. In the public sector, subordinates' trust in leaders is the basis for the practice of leaders' ISB. Without this basis, leaders' information-sharing effect would be greatly weakened. This study found that ET, as a boundary condition affecting civil servants' TCB, has a significant moderated effect. We suggested that leaders and subordinates in their usual work are consistent with words and deeds and establish a reliable and trustworthy image of each other. Therefore, leaders can deepen the interaction and communication with subordinates in work, life, and other aspects. In this way, ET would be cultivated, and then subordinates' TCB would have occurred easier.

### Limitation of study

The limitations of this study are as follows: First, this study only focuses on the individual level of civil servants and takes PSM as a mediated variable to explore the influence path of leaders' ISB in the public sector, which is slightly inadequate in the model explanation. In future studies, the factors influencing subordinates' TCB at the organizational level, such as organizational atmosphere, can be added to the model to increase the explanation. Second, in terms of data collection, the measurement of core variables in this study adopts civil servants' self-assessment and is carried out in the same period, so it is difficult to further determine the causal relationship of the model. In future studies, time series design and other evaluations will be used to collect data to reduce the deviation of common methods. Although we believe that the sample size should be 5–10 times the items in the scale, and 200 copies have reached the minimum standard of the sample size, it is still necessary to expand the sample size in future research.

## Data availability statement

The raw data supporting the conclusions of this article will be made available by the authors, without undue reservation.

## Author contributions

J-NL and Y-ZH: conceptualization, writing—original draft, writing—review and editing, methodology, and investigation. JW: conceptualization, writing—review and editing, supervision, formal analysis, and methodology. C-ZX and PF: writing—review and editing, investigation, and validation. All authors reviewed and approved this article for publication.

## References

[B1] Ahmad A. B. Liu B. C. Butt A. S. (2020). Scale development and construct clarification of change recipient proactivity. Person. Rev. 49, 1619–1635. 10.1108/PR-02-2019-0091

[B2] Aiken L. S. West S. G. (1991). Multiple Regression: Testing and Interpreting Interactions. Newbury Park, CA: Sage Press

[B3] Ajzen I. (1991). The theory of planned behavior. Organ. Behav. Hum. Dec. Process. 50, 179–211.

[B4] Ajzen I. (2011). The theory of planned behavior: reactions and reflections. Psychol. Health 26, 1113–1127. 10.1080/08870446.2011.613995 21929476

[B5] Bao Y. J. Li C. P. (2016). Measurement of public service motivation: Theoretical structure and scale revision. Human Resour. Develop. China. 7, 83–91. 10.16471/j.cnki.11-2822/c.2016.07.010

[B6] Bass B. M. (1985). Leadership and Performance Beyond Expectations. New York: Free Press

[B7] Bettencourt L. A. (2004). Change-oriented organizational citizenship behaviors: the direct and moderating influence of global orientation. J. Retail. 80, 165–180. 10.1016/j.jretai.2003.12.001

[B8] Borins S. (2000). Loose cannons and rule breakers, or enterprising leaders? Some evidence about innovative public managers. Public Admin. Rev. 60, 498–507. 10.1111/0033-3352.00113

[B9] Bysted R. Hansen J. R. (2015). Comparing public and private sector employees' innovative behavior: understanding the role of job and organizational characteristics, job types and subjectors. Public Manag. Rev. 17, 698–717. 10.1080/14719037.2013.841977

[B10] Chan S. C. (2014). Paternalistic leadership and employee voice: does information sharing matter. Hum. Relat. 67, 667–693. 10.1177/0018726713503022

[B11] Chen D. X. Liu B. C. (2013). A study on the formation mechanism of grassroots civil servants' transformational responsibility behavior: the trickle-down effect of public service motivation. Public Manag. Rev. 1, 47–67.

[B12] Chen D. X. Liu B. C. (2021). An empirical study on the impact of ethical leadership on change-oriented organizational citizenship behavior of grassroots civil servants. J. Central South Univ. 27, 100–111.

[B13] Chen D. X. Liu B. C. Long T. Y. (2019). Analysis of civil servant turnover intention based on embedded perspective. J. Shanghai Jiaotong Univ. 27, 120–129.

[B14] Chen Q. Q. Fan Y. Lv X. Li C. X. (2018). The influence mechanism of leaders' information sharing behavior on employee performance: the mediating role of job engagement and the moderating role of emotional trust. Prediction 3, 15–21.

[B15] Chen X. P. Eberly M. B. Chiang T. J. Farh J. L. Cheng B. S. (2014). Affective trust in Chinese leaders: linking paternalistic leadership to employee performance. J. Manag. 40, 796–819. 10.1177/0149206311410604

[B16] Chen Z. Wu H. (2008). Intrinsic motivation and its antecedent variables. Adv. Psychol. Sci. 1, 98–105.

[B17] Chen Z. M. Lin Y. Q. (2016). Does the leader-relational behavior of government departments influence the transformational organizational citizenship behavior of subordinates? The mediating role of public service motivation and the moderating role of organizational support. J. Public Manag. 13, 11–20+152.

[B18] Chiaburu D. S. Baker V. L. (2006). Extra-role behaviors challenging the status-quo: validity and antecedents of taking charge behaviors. J. Manag. Psychol. 21, 620–637. 10.1108/02683940610690178

[B19] Damanpour F. Schneider M. (2009). Characteristics of innovation and innovation adoption in public organizations: assessing the role of managers. J. Public Admin. Res. Theory 19, 495–522. 10.1093/jopart/mun021

[B20] Demircioglu M. A. (2018). The effects of empowerment practices on perceived barriers to innovation: evidence from public organizations. Int. J. Public Admin. 41, 1302–1313. 10.1080/01900692.2017.1387143

[B21] Demircioglu M. A. (2020). The effects of organizational and demographic context for innovation implementation in public organizations. Public Manag. Rev. 22, 1852–1875. 10.1080/14719037.2019.1668467

[B22] Duan W. T. Jiang G. G. (2008). A review of planned behavior theory. Adv. Psychol. Sci. 16, 315–320.

[B23] Fattore G. Iacovone D. Steccolini I. (2018). Managing successful change in the public sector: a view from the consultants' world. Public Manag. Rev. 20, 587–606. 10.1080/14719037.2017.1340504

[B24] Fernandez S. Moldogaziev T. (2013). Using employee empowerment to encourage innovative behavior in the public sector. J. Public Admin. Res. Theory 23, 155–187. 10.1093/jopart/mus008

[B25] Frederickson H. G. Johnston J. M. eds. (1999). Public Management Reform and Innovation: Research, Theory and Application. Tuscaloosa: University of Alabama Press.

[B26] Gao L. Janssen O. Shi K. (2011). Leader trust and employee voice: the moderating role of empowering leader behaviors. Lead. Q. 22, 787–798. 10.1016/j.leaqua.2011.05.015

[B27] Ge L. L. (2016). The impact of transformational leadership on civil servants' work attitude: the mediating effect of public service motivation. J. Yantai Univ. 29, 111–120.

[B28] Guo Y. Liao J. Liao S. Zhang Y. (2014). The mediating role of intrinsic motivation on the relationship between developmental feedback and employee job performance. Soc. Behav. Person. Int. J. 42, 731–741. 10.2224/sbp.2014.42.5.731

[B29] Guo Y. Liao J. Q. (2014). Research on the mechanism of supervisory developmental feedback on employee performance. Manag. Sci. 27, 99–108.

[B30] Hackman J. R. Oldham G. R. (1976). Motivation through the design of work: test of a theory. Organ. Behav. Hum. Perform. 16, 250–279.

[B31] Hansen J. A. Pihl-Thingvad S. (2019). Managing employee innovative behavior through transformational and transactional leadership styles. Public Manag. Rev. 21, 18–944. 10.1080/14719037.2018.1544272

[B32] Hao P. Long L. R. (2020). Transforming passivity into initiative: the impact and mechanism of shared leadership on employees' proactive change behavior. J. Manag. Eng. 34, 11–20.

[B33] Hassan H. A. Zhang X. D. Ahmad A. B. Liu B. (2021). Public service motivation and employee change-supportive intention: utilizing the theory of planned behavior. Public Person. Manag. 50, 283–304. 10.1177/0091026020934515

[B34] Hassan S. (2015). The Importance of ethical leadership and personal control in promoting improvement-centered voice among government employees. J. Public Admin. Res. Theory 25, 697–719. 10.1093/jopart/muu055

[B35] Hatfield J. D. Huseman R. C. (1982). Perceptual congruence about communication as related to satisfaction: moderating effects of individual characteristics. Acad. Manag. J. 25, 349–358.

[B36] Hayes A. F. (2013). Introduction to mediation, moderation, and conditional process analysis: a regression-based approach. J. Educ. Meas. 51, 335–337. 10.1111/jedm.12050

[B37] He P. Jiang C. Xu Z. Shen C. (2021). Knowledge hiding: current research status and future research directions. Front. Psychol. 12, 748237. 10.3389/fpsyg.2021.748237 34777143 PMC8586422

[B38] He P. Peng Z. Zhao H. Estay C. (2019). How and when compulsory citizenship behavior leads to employee silence: a moderated mediation model based on moral disengagement and supervisor–subordinate guanxi views. J. Bus. Ethics 155, 259–274. 10.1007/s10551-017-3550-2

[B39] He P. Sun R. Zhao H. Zheng L. Shen C. (2020). Linking work-related and non-work-related supervisor–subordinate relationships to knowledge hiding: a psychological safety lens. Asian Bus. Manag. 21, 1–22. 10.1057/s41291-020-00137-9

[B40] Homberg F. Vogel R. Weiherl J. (2019). Public service motivation and continuous organizational change: taking charge behavior at police services. Public Admin. 97, 28–47. 10.1111/padm.12354

[B41] Kamarck E. C. (2003). Government Innovation around the World, Ash Institute for Democratic Governance and Innovation. Harvard: John F. Kennedy School of Government, Harvard University.

[B42] Kim T. Y. Liu Z. Q. Diefendorff J. M. (2015). Leader-member exchange and job performance: the effects of taking charge and organizational tenure. J. Organ. Behav. 36, 216–231. 10.1002/job.1971

[B43] Li F. Wang P. Q. (2016). The structure and antecedent analysis of the motivation of grassroots public service. J. Central China Normal Univ. 55, 29–38.

[B44] Li J. P. (2004). Public Service Government. Beijing: Peking University Press.

[B45] Li M. Rong Y. Li R. (2019). Responsibility for change in organizations: a new topic in the study of positive organizational behavior. Psychol. Sci. 42, 715–721.

[B46] Lin Y. Q. Zhang Y. Q. (2019). Will leading member exchange influence civil servants' transformational organizational citizenship behavior? The mediating role of sense of obligation to change and the moderating role of public service motivation. Public Admin. Rev. 1, 132–150.

[B47] Liu B. Hu W. Chen G. Y. (2015). From the west to the east: validating servant leadership in the Chinese public sector. Public Person. Manag. 44, 25–45. 10.1177/0091026014555995

[B48] Liu B. C. (2015). Research on Public Service Motivation in the Context of China. Shanghai: Shanghai Jiao Tong University Press.

[B49] Liu B. C. Perry J. L. Tan X. Y. Zhou X. (2018). A cross-level holistic model of public service motivation. Int. Public Manag. J. 21, 703–728. 10.1080/10967494.2017.1370046

[B50] Mcallister D. (1995). Affect-based and cognition-based trust as foundations for interpersonal cooperation in organizations. Acad. Manag. J. 38, 24–59.

[B51] Meijer A. J. (2014). From hero-innovators to distributed heroism: an in-depth analysis of the role of individuals in the public sector innovation. Public Manag. Rev. 16, 199–216. 10.1080/14719037.2013.806575

[B52] Miao Q. Newman A. Huang X. (2014). The impact of participative leadership on job performance and organizational citizenship behavior: distinguishing between the mediating effects of affective and cognitive trust. Int. J. Hum. Resour. Manag. 25, 2796–2810. 10.1080/09585192.2014.934890

[B53] Miao Q. Newman A. Schwarz G. Cooper B. (2018). How leadership and public service motivation enhance innovation behavior. Public Admin. Rev. 78, 71–81. 10.1111/puar.1283935731943

[B54] Nanjing University of Administration Research Group (2004). Leadership view in the information network era. J. Nanjing Munic. Party School Nanjing Admin. College 4, 34–37.

[B55] Nifadkar S. S. Wu W. Gu Q. (2019). Supervisors' work-related and non-work information sharing: integrating research on information sharing, information seeking, and trust using self-disclosure theory. Person. Psychol. 72, 241–269. 10.1111/peps.12305

[B56] Perry J. L. (1996). Measuring public service motivation: an assessment of construct reliability and validity. J. Public Admin. Res. Theory 6, 5–22.

[B57] Perry J. L. (2000). Bringing society in: toward a theory of public service motivation. J. Public Admin. Res. Theory 10, 471–488. 10.1093/oxfordjournals.jpart.a024277

[B58] Perry J. L. Hondeghem A. Wise L. R. (2010). Revisiting the motivational bases of public service: 20 years of research and an agenda for the future. Public Admin. Rev. 70, 681–690. 10.1111/j.1540-6210.2010.02196.x

[B59] Perry J. L. Wise L. R. (1990). The motivational bases of public service. Public Admin. Rev. 50, 367–373

[B60] Pundt A. (2015). The relationship between humorous leadership and innovative behavior. J. Manag. Psychol. 30, 878–893. 10.1108/JMP-03-2013-0082

[B61] Shi K. Gao L. P. Huang X. Sha Y. J. (2012).The effect of leadership empowerment on employee silence: the moderating role of trust. Manag. Rev. 24, 94–101.

[B62] Snyder R. A. Morris J. H. (1984). Organizational communication and performance. J. Appl. Psychol. 69, 461–465.

[B63] Tan X. Y. (2018). Research on the impact mechanism of multi-level situational variables on grassroots civil servants' innovation behavior from the perspective of planned behavior. Chin. J. Manag. 7, 1001–1011.

[B64] Tan X. Y. (2019). External environment change, service motivation and grassroots civil servants' reform behavior: a mixed study based on the survey of grassroots civil servants in four provinces in China. Public Admin. Rev. 12, 63–84.

[B65] Tang J. Fang Z. B. (2020). The influence mechanism of service-oriented leadership on civil servant innovation behavior. J. Shanghai Jiaotong Univ. 4, 88–98.

[B66] Tian Q. Sanchez J. I. (2017). Does paternalistic leadership promote innovative behavior? The interaction between authoritarianism and benevolence. J. Appl. Soc. Psychol. 47, 235–246. 10.1111/jasp.12431

[B67] Torugsa N. Arundel A. (2015). The nature and incidence of workgroup innovation in the Australian public sector: evidence from the Australian 2011 state of the service survey. Aust. J. Public Admin. 75, 202–221. 10.1111/1467-8500.12095

[B68] Vandervoet J. (2016). Change leadership and public sector organizational change: examining the interactions of transformational leadership style and red tape. Am. Rev. Public Admin. 46, 660–682. 10.1177/0275074015574769

[B69] Vigoda-Gadot E. Beeri I. (2012). Change-oriented organizational citizenship behavior in public administration: the power of leadership and the cost of organizational politics. J. Public Admin. Res. Theory 22, 573–596. 10.1093/jopart/mur036

[B70] Wang Y. H. Shu Q. F. (2018). Public service motivation of rural cadres in China: quantitative measurement and influencing factors. Manag. World 34, 93–102.

[B71] Wei H. M. Long L. R. (2011). The influence of cognitive and emotional trust, power distance and institutional control on leadership empowerment behavior. J. Manag. Eng. 25, 10–17.

[B72] Wu Z. M. Wu X. (2007). The relationship between transformational leadership, organizational citizenship behavior and psychological empowerment. J. Manag. Sci. 5, 40–47.32599719

[B73] Wynen J. Boon J. Kleizen B Verhoest K. (2020). How multiple organizational changes shape managerial support for innovative work behavior: evidence from the Australian public service. Rev. Public Person. Admin. 40, 491–515. 10.1177/0734371X18824388

[B74] Xu S. Y. Ou Y. K. (2012). The influence of leadership justice and information justice on organizational retaliation behavior based on reference cognitive theory. J. Manag. 9, 1457–1463.

[B75] Zada M. Zada S. Ali M. Jun Z. Y. Contreras-Barraza N. Castillo D. (2022a). How classy servant leader at workplace? Linking servant leadership and task performance during the COVID-19 crisis: a moderation and mediation approach. Front. Psychol. 13, 810227–810227. 10.3389/fpsyg.2022.810227 35401384 PMC8984191

[B76] Zada M. Zada S. Khan J. Saeed I. Zhang Y. J. Vega-Muñoz A.. (2022b). Does servant leadership control psychological distress in crisis? Moderation and mediation mechanism. Psychol. Res. Behav. Manag. 15, 607. 10.2147/PRBM.S354093 35310833 PMC8926009

[B77] Zhang J. Liu L. A. Liu W. (2015). Trust and deception in negotiation: culturally divergent effects. Manag. Organ. Rev. 11, 123–144. 10.1111/more.12028

[B78] Zhang J. Zhu J. B. Li Y. Edward L. D. (2010). An effective approach to promoting job motivation: a perspective of self-determination theory. Adv. Psychol. Sci. 18, 752–759.

[B79] Zhang X. Zhou J. (2014). Empowering leadership, uncertainty avoidance, trust, and employee creativity: interaction effects and a mediating mechanism. Organ. Behav. Hum. Dec. Process. 124, 150–164. 10.1016/j.obhdp.2014.02.002

[B80] Zhang Z. T. Zhao L. J. Ding M. Z. (2020). The influence mechanism of empowering leadership on employees' proactive change behavior. Sci. Res. Manag. 41, 218–226.

[B81] Zhu X. X. Li C. Wang X. L. Liu J. N. Xia S. (2021). How does information sharing of a supervisor influence proactive change behavior of an employee? The chain mediating role of family-like employee–organization relationship and relationship energy. Front. Psychol. 12, 2617. 10.3389/fpsyg.2021.739968 35002839 PMC8735874

